# The enhanced expression of death receptor 5 (DR5) mediated by HBV X protein through NF-kappaB pathway is associated with cell apoptosis induced by (TNF-α related apoptosis inducing ligand) TRAIL in hepatoma cells

**DOI:** 10.1186/s12985-015-0416-z

**Published:** 2015-11-17

**Authors:** Fanyun Kong, Hongjuan You, Jinjin Zhao, Wen Liu, Lei Hu, Wenya Luo, Wei Hu, Renxian Tang, Kuiyang Zheng

**Affiliations:** Department of Pathogenic Biology and Immunology, Laboratory of Infection and Immunity, Xuzhou Medical College, 209 Tongshan Road, Xuzhou, Jiangsu 221004 China

**Keywords:** HBX, Apoptosis, TRAIL, DR4, DR5

## Abstract

**Background:**

HBV X protein (HBX) is associated with cell apoptosis mediated by TNF-α related apoptosis inducing ligand (TRAIL), while the role of HBX on the expressions of TRAIL receptors death receptor 4 (DR4) and DR5 are unclear. In this study, we detected the cell apoptosis induced by TRAIL as well as gene and protein expressions of DR4 and DR5 in Huh-7 cells steadily transfected with HBX (Huh-7-HBX cells). In addition, we investigated the activation of different pathways associated with the expressions of TRAIL receptors in Huh-7-HBX cells.

**Methods:**

The apoptosis of Huh-7-HBX cells induced by TRAIL was evaluated by flow cytometry analysis. The levels of DR4 and DR5 expression in cells were determined by real-time PCR and western blotting analysis. The activities of JNK pathway and NF-kappaB (NF-κB) pathway were demonstrated by western blotting assay.

**Results:**

Compared to control cells, the percentage of cell apoptosis was increased in Huh-7-HBX cells. The increased expressions of DR4 and DR5 on gene and protein levels were observed in Huh-7-HBX cells. Further researches suggested that activation of JNK pathway was increased but not involved in the expression of TRAIL receptors in HBX positive cells. The activation of NF-κB pathway increased and was responsible for DR5 expression and cell apoptosis in HBX positive cells.

**Conclusions:**

These results demonstrate that increased apoptosis induced by TRAIL is associated with increased expression of DR5 that mediated by HBX through NF-κB pathway. This finding provides a critical insight into the mechanism of hepatocyte apoptosis mediated by HBX in HBV infection.

## Background

Hepatitis B virus (HBV) infection is responsible for chronic hepatitis, cirrhosis and hepatocellular carcinoma (HCC) [1]. Among the proteins encoded by the viral genome, hepatitis B virus X (HBX) protein has been found to play a crucial role in pathogenesis of HCC. Accumulating evidences suggest that HBX protein not only could activate transcription factors, such as nuclear factor-κB (NF-κB) and activator protein 1 (AP-1) [2–4], but also interact with different cellular oncogenes, including Ras and Src to enhance cell proliferation, migration, invasion and metastasis [5, 6]. HBX also has the function of stimulating Wnt, Ras/MAPK, and PI3K-Akt/PKB pathway to mediate the expression of cellular proteins with various biological activities [7, 8]. In addition, our laboratory and other researchers reported that HBX was involved in mediating cell apoptosis by the death receptor pathway or mitochondrial dependent pathway [9–12].

TNF-α related apoptosis inducing ligand (TRAIL) is a member of the TNF (tumor necrosis factor) superfamily that can induce cell apoptosis mainly in tumor cells. Until now, five receptors of TRAIL have been reported, but only death receptors TRAIL-R1 (DR4) and TRAIL-R2 (DR5) have cytoplasmic death domains to induce cell apoptosis. TRAIL-R3, TRAIL-R4 and osteoprotegerin lack the functional death domains but are capable of blocking apoptosis mediated by TRAIL [13]. After the binding of TRAIL with death receptors, caspase-8 and caspase-10 are recruited and form a death-inducing signaling complex (DISC) to transduce apoptotic signal. Current studies reported that HBX could increase TRAIL induced apoptosis by up-regulating of Bax protein or increasing TRAIL expression in hepatoma cells [14, 15]. But the role of TRAIL receptors in HBX-mediated apoptosis through TRAIL is largely unknown.

In this study, we propose that the HBX maybe induce the abnormal expression of death receptors to participate in cell apoptosis induced by TRAIL. We assessed the expression of DR4 and DR5 in HBX positive hepatoma cells and provided a new insight into the mechanism of DR5 expression mediated by HBX.

## Results

### The effect of HBX on cell apoptosis induced by TRAIL

**Fig. 1:**
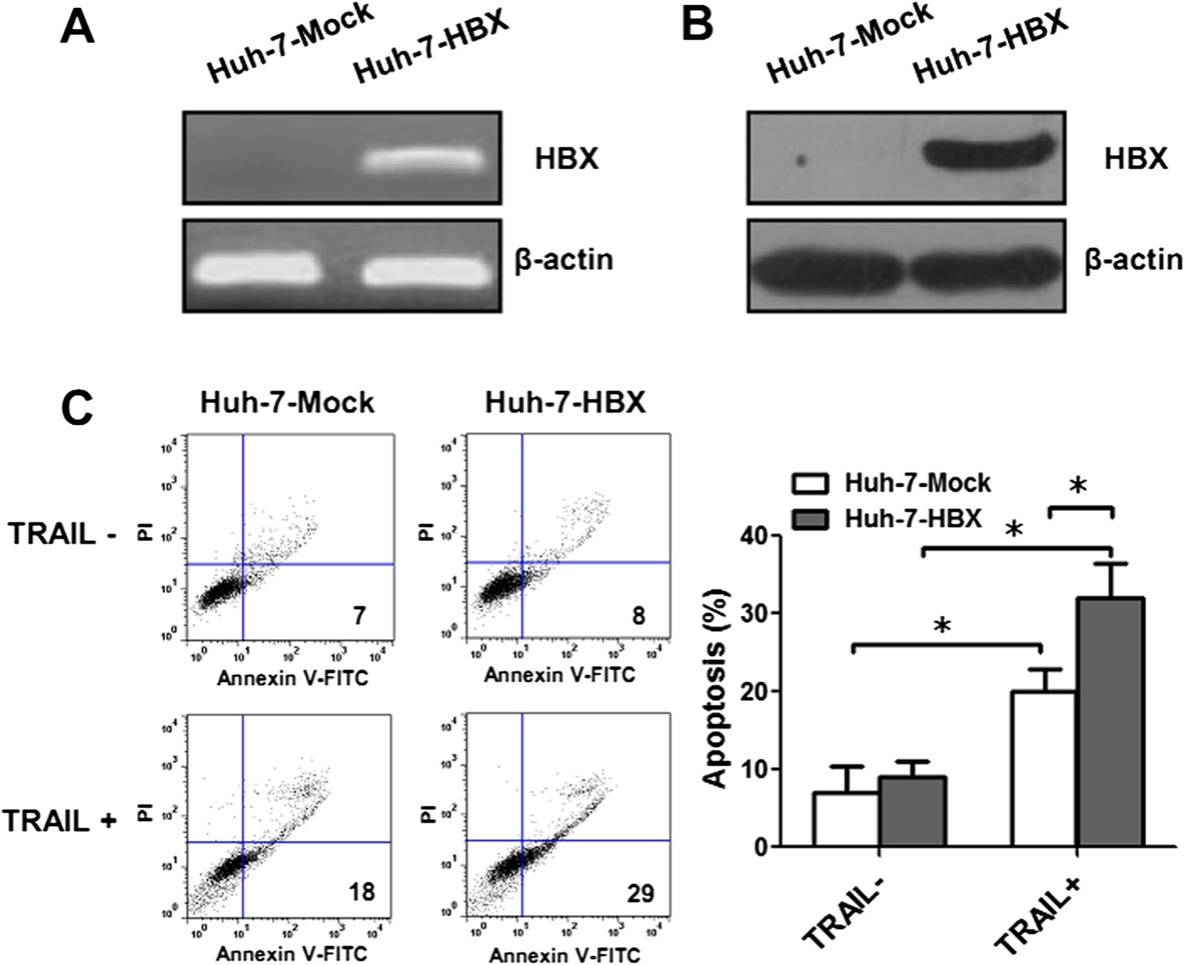
The apoptosis of Huh-7-HBX cells induced by TRAIL. **a**, **b** The expression of HBX in HepG2 cells was determined by RT-PCR and western blotting analysis. **c** After the cells treated with 100 ng/mL TRAIL for 24 h, the detection of apoptosis of Huh-7-HBX cells and control cells by flow cytometry

To investigate the role HBX on apoptosis of hepatoma cell line Huh-7 cell with the induction of TRAIL, HBX expressing plasmid pcDNA3.1-X was transfected into Huh-7 cells (named as Huh-7-HBX cells). The Huh-7 cells transfected with plasmid pcDNA3.1 was used as control cells (Huh-7-Mock cells). Real-time PCR and western blotting analysis demonstrated that Huh-7 cells transfected with pcDNA3.1-X could steadily express HBX gene and protein (Fig. 1a-b). Then Huh-7-HBX cells and control cells were incubated with TRAIL for 24 h, and the cell apoptosis was detected by flow cytometry. The results showed that the percentage of cell apoptosis of Huh-7-HBX cells was significant higher than that of control cells (Fig. 1c), suggesting that HBX could promote the cell apoptosis induced by TRAIL.

### The role of HBX on induction of death receptors expression

**Fig. 2:**
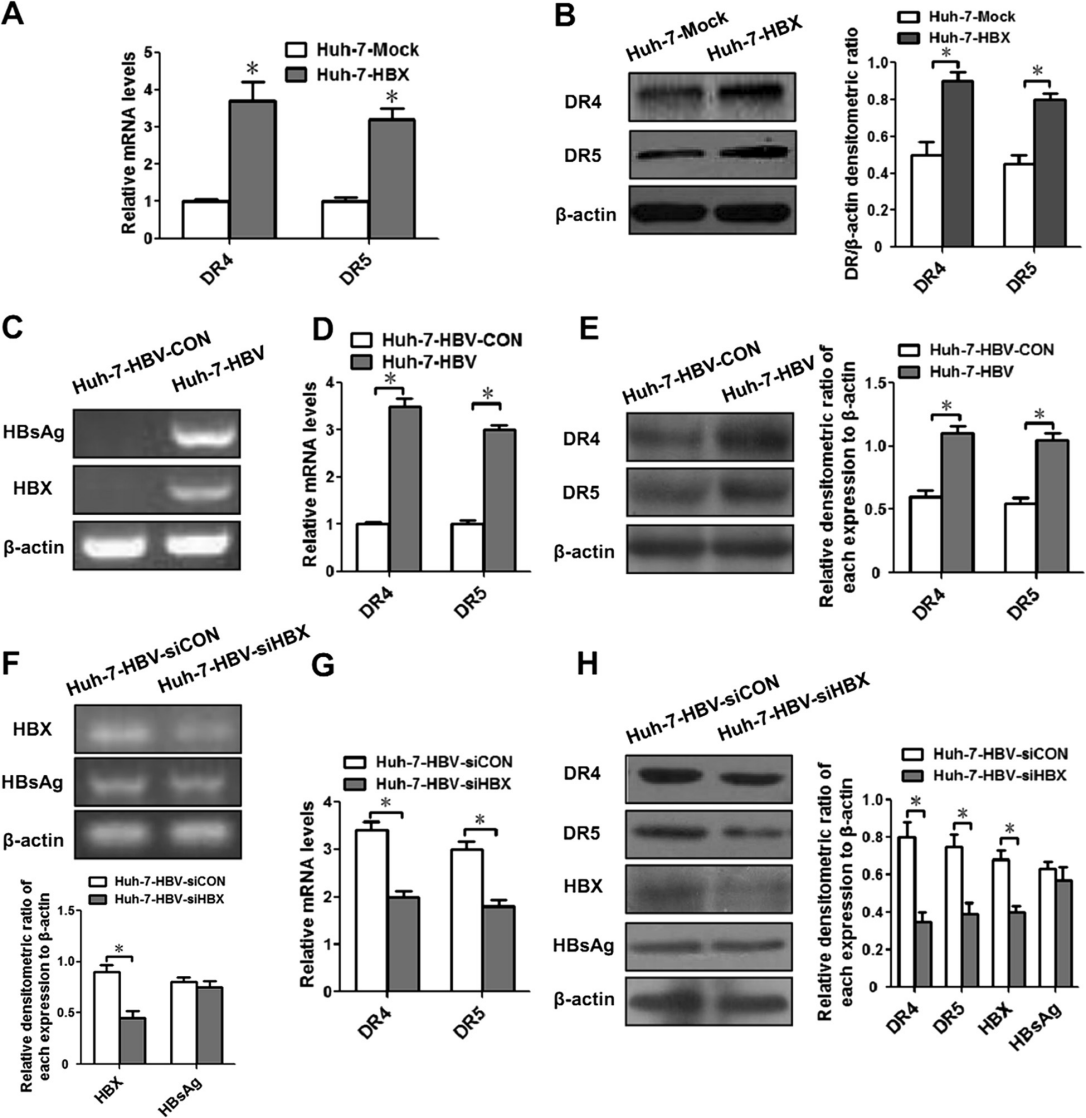
The expressions of DR4 and DR5 mediated by HBX. **a** The expressions of DR4 and DR5 mRNA detected by real-time PCR in Huh-7-HBV cells and control cells. **b** The expressions of DR4 and DR5 proteins determined by western blotting analysis in Huh-7-HBV cells and control cells. To detect the role of HBV on DR4 and DR5 expressions, Huh-7 cells were transfected with pUC18-HBV1.2 or control plasmid. Forty-eight hours after transfection, the expressions of HBX and HBsAg genes (**c**), the expressions of DR4 and DR5 were detected on mRNA levels (**d**) and protein levels (**e**) were measured. The Huh-7 cells transfected with pUC18-HBV1.2 was named as Huh-7-HBV cells, and the cells transfected with control plasmid was as Huh-7-HBV-CON cells. In order to test the role of HBX on the expressions of DR4 and DR5 mediated by HBV, Huh-7 cells were cotransfected pUC18-HBV1.2 with pSilencer3.1-shHBX or control plasmid. Forty-eight hours after transfection, the expressions of HBX, HBsAg as well as DR4 and DR5 were detected on mRNA levels with detected by RT-PCR (**f**) and real-time PCR (**g**), and protein levels measured by western blotting analysis (**h**). The Huh-7 cells cotransfected with pUC18-HBV1.2 with pSilencer3.1-shHBX were named as Huh-7-HBV-siHBX cells. The Huh-7 cells cotransfected with pUC18-HBV1.2 with control plasmid of pSilencer3.1-shHBX were as Huh-7-HBV-siCON cells

Considering that the cell apoptosis induced by TRAIL was based on the interaction of TRAIL with the receptors DR4 and DR5, we detected the expressions of DR4 and DR5 on gene and protein levels in Huh-7-HBX cells and control cells. The results showed that, compared with control cells, not only the gene expression of DR4 and DR5 but also protein expression of these receptors were significant higher in HBX-Huh-7 cells than in control cells (Fig. 2a-b), suggesting that HBX could upregulate the expressions of DR4 and DR5 in hepatoma cells. In order to explore whether HBV could mediate the expressions of DR4 and DR5, we transfected the pUC18-HBV1.2, a plasmid contains the full-length HBV DNA, into Huh-7 cells (named as Huh-7-HBV cells) (Fig. 2c), and the results showed increased expressions of DR4 and DR5 on mRNA and protein levels in HBV positive cells (Fig. 2d-e). Furthermore, when the pUC18-HBV-1.2 plasmid and pSilencer3.1-shHBX, a plasmid could inhibit the expression of HBX, were cotransfected into Huh-7 cells (named as Huh-7-HBV-siHBX) (Fig. 2f), the expressions of DR4 and DR5 were decreased (Fig. 2g-h). Taken together, these results suggested that the increased expressions of DR4 and DR5 induced by HBV were mainly dependent on HBX in Huh-7 cells.

### The effect of different pathways on the DR4 and DR5 expression mediated by HBX

**Fig. 3:**
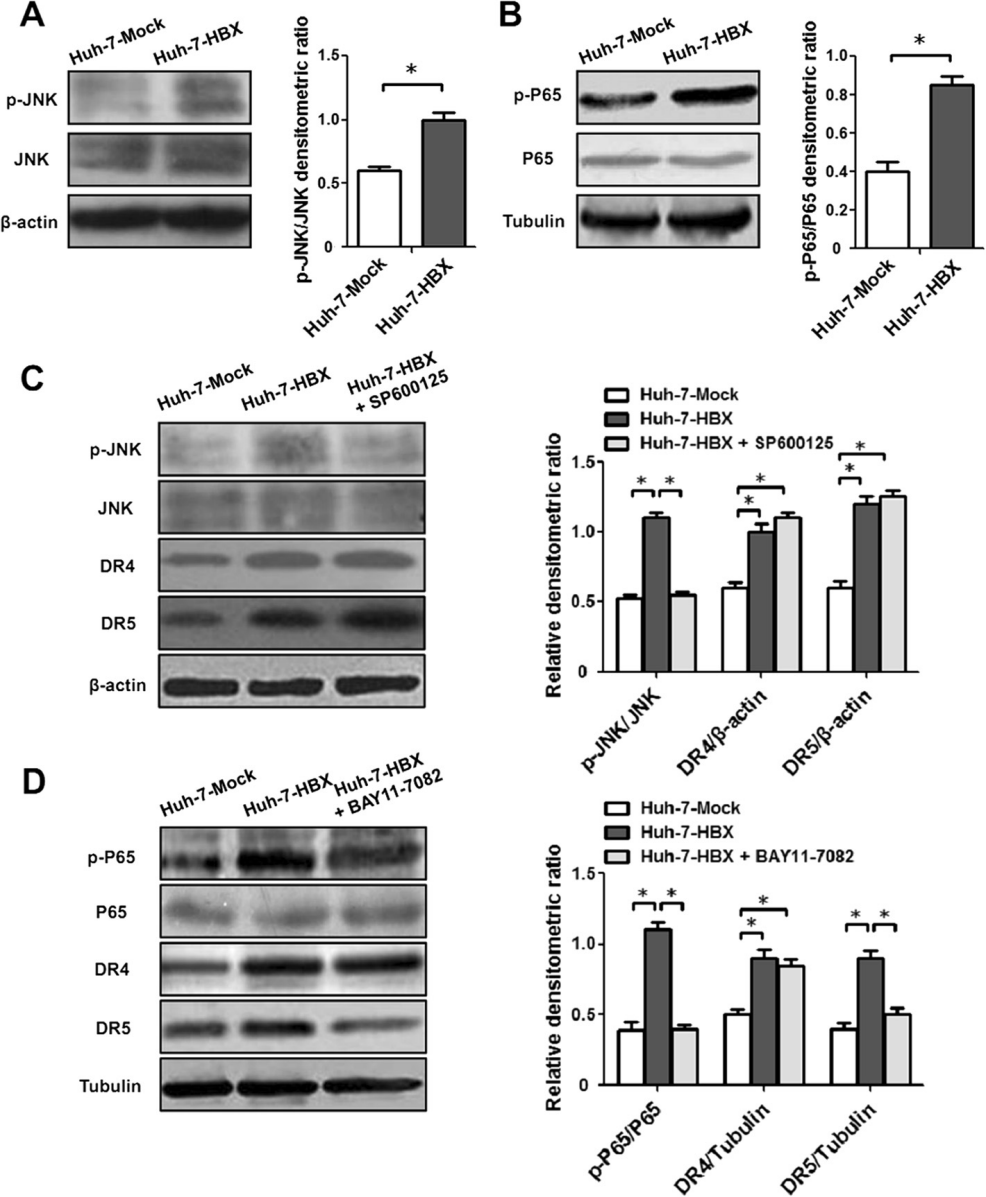
The roles of JNK and NF-κB pathways on expressions of DR4 and DR5 proteins in Huh-7-HBX cells. **a** The activation of JNK pathways induced by HBX. **b** The activation of NF-κB pathways induced by HBX. **c** The role of JNK pathways on expression of DR4 and DR5 proteins induced by HBX. Huh-7-HBX and control cells were cultured in 0.01 % dimethyl sulfoxide (DMSO) in the absence or presence of 20 μM SP600125 for 24 h. Then the expressions of DR4 and DR5 proteins were detected by western blotting analysis. **d** The role of NF-κB pathways on expressions of DR4 and DR5 proteins induced by HBX. Huh-7-HBX and control cells were cultured in 0.01 % dimethyl sulfoxide (DMSO) in the absence or presence of 10 μM BAY11-7082 for 24 h, and then the expressions of DR4 and DR5 proteins were detected by western blotting analysis

Pervious researches showed that the expressions of DR4 and DR5 were mediated by different pathways such as JNK pathway and NF-kappa B (NF-κB) pathway [16–19]. We measured the phosphorylation levels of JNK in Huh-7-HBX cells and control cells. Compared to control cells, increased phosphorylation level of JNK was observed in Huh-7-HBX cells (Fig. 3a). Then we treated the cells with JNK inhibitor (SP600125) for 24 h. The results indicated that though SP600125 could inhibit the levels of JNK phosphorylation in Huh-7-HBX cells, the expression of DR4 and DR5 on mRNA and protein levels had no significant change in the HBX positive cells (Fig. 3c). These results suggested that activation of JNK pathway could be mediated by HBX, but it had no role on expressions of DR4 and DR5 in Huh-7-HBX cells.

We next detected the role of NF-κB pathway on expressions of DR4 and DR5 in Huh-7-HBX cells. The result showed that the activation of NF-κB (phosphorylation of P65) pathway increased in Huh-7-HBX cells, compared to control cells (Fig. 3b). When treated the cells with NF-κB inhibitor (BAY11-7082) for 24 h, the activation of NF-κB pathway declined in HBX-Huh-7 cells (Fig. 3d). Meanwhile, the expression of DR5 but not DR4 also decreased in HBX-Huh-7 cells, suggesting the NF-κB pathway played an important role on expression of DR5 mediated by HBX in hepatoma cells.

### The role of NF-κB pathway on cell apoptosis mediated by TRAIL in Huh-7-HBX cells

**Fig. 4:**
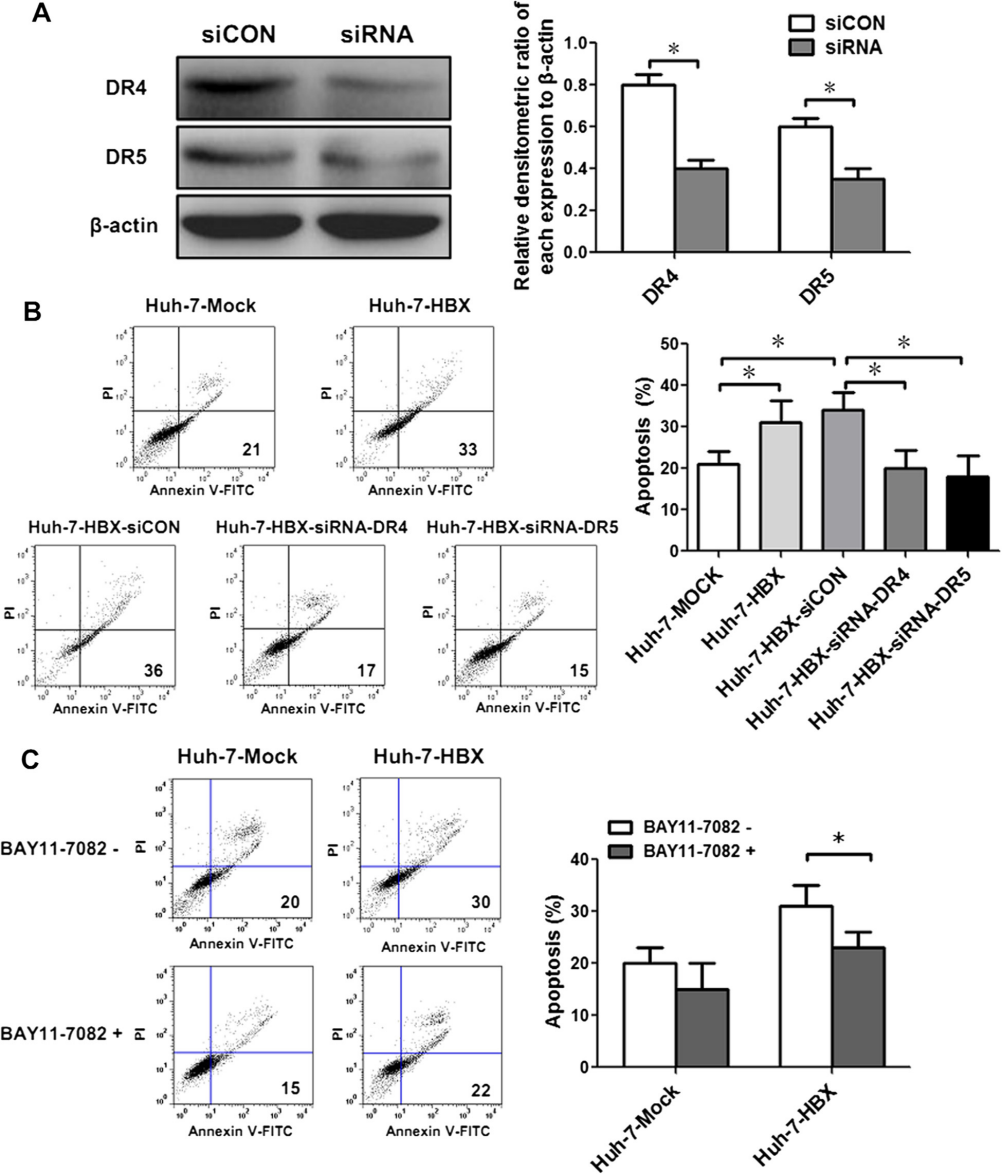
The role of NF-κB pathways on apoptosis of Huh-7-HBX cells induced by TRAIL. **a** Huh-7-HBX cells was transfected with shRNA plasmids for DR4 and DR5, and 48 h post-transfection, the expression of DR4 and DR5 proteins was measured by western blotting analysis. Huh-7-HBX cells transfected with control plasmid of shRNA were named as siCON cells, Huh-7-HBX cells transfected with shRNA plasmids for DR4 and DR5 (pGPU6/GFP/Neo-DR4 and pGPU6/GFP/Neo-DR5) were as siRNA cells. **b** After Huh-7-HBX cells transfected with shRNA plasmids for DR4 and DR5, and control plasmids for 48 h, the apoptosis was detected in Huh-7-Mock cells, Huh-7-HBX cells, and Huh-7-HBX cells transfected with different plasmids. **c** After the cells treated with 100 ng/mL TRAIL and 10 μM BAY11-7082 for 24 h, the apoptosis of cells was detected by flow cytometry analysis

In order to investigate whether the apoptosis of Huh-7-HBX cells mediated by TRAIL was dependent on DR4 and DR5, we constructed the specific short hairpin RNA (shRNA) vectors for DR4 and DR5. When transfected these shRNA vectors into Huh-7-HBX cells, the expressions of DR4 and DR5 proteins were significantly inhibited (Fig. 4a). Moreover, the apoptosis of Huh-7-HBX cells was decreased when transfection of shRNA plasmids for DR4 and DR5, compared to Huh-7-HBX cells and Huh7-HBX cells transfected with control vectors (Fig. 4b). Taken together, these results indicated that the apoptosis induced by TRAIL in Huh-7-HBX cells was mainly relied on DR4 and DR5.

Given that NF-κB pathway is responsible for the expression of DR5 in Huh-7-HBX cells, we next detected the role of NF-κB pathway on apoptosis of Huh-7-HBX cells treated with TRAIL. As in Fig. 4c shown, when cells co-treated with TRAIL and BAY11-7082 for 24 h, the rates of cell apoptosis decreased in HBX-Huh-7 cells. While the BAY11-7082 did not have a significant influence on apoptosis of control cells induced by TRAIL. Taken together, these results suggested that the NF-κB pathway contributed to the cell apoptosis mediated by TRAIL in Huh-7-HBX cells.

## Discussion

The cell apoptosis induced by TRAIL have been investigated in HBX positive hepatocytes or hepatoma cells. While the role of TRAIL receptors on HBX-mediated apoptosis had not be well identified. In this study, we found the upregulation of DR4 and DR5 mediated HBX was associated with apoptosis of hepatoma cells treated with TRAIL. In addition, the activation of NF-κB was responsible for the expression of DR5 mediated by HBX and promoted the cell apoptosis induced by TRAIL in HBX positive hepatoma cells.

Consistent with previous researches, we found that HBX sensitized the cells to apoptosis induced by TRAIL. Liang X et al reported that HBX strengthened TRAIL-induced apoptosis of hepatocytes through increasing the expression of Bax, a protein associated with cell apoptosis mediated by mitochondria-dependent pathway [14]. In addition, Yang Y et al showed that the apoptosis mediated by HBX was associated with increased expression of TRAIL induced by HBX in hepatocytes [15]. While the apoptosis mediated by TRAIL was based on the binding of TRAIL with its receptors. The apoptosis mediated by HBX in hepatocytes maybe associated with TRAIL receptors. In addition, increased expression of DR4 was observed in hepatoma cell line HepG2 transfected with HBV by Janssen HL et al [20], and the observation enhance our hypothesis that HBX could increase the sensitivity of cell apoptosis induced by TRAIL receptors. Among the TRAIL receptors, only DR4 and DR5 have cytoplasmic death domains and induce cell apoptosis by interacting with TRAIL. We tested the expressions of DR4and DR5 in HBX positive hepatoma cells and the results were consistent with our hypothesis that HBX could induce DR4 and DR5 expression in Huh-7-HBX cells. In addition, we found that HBV was capable of inducing the expressions of DR4 and DR5, and HBX was responsible for the increased expressions of DR4 and DR5 mediated by HBV.

We next investigated the mechanism of increased expression of DR4 and DR5 induced by HBX. Previous studies indicated that expressions of DR4 and DR5 could be mediated by JNK-dependent pathway [16, 17]. Consistent with our previous researches, we observed increased phosphorylation levels of JNK in HBX positive cells [9]. But when we inhibit JNK-dependent pathway with SP600125, the expressions of DR4 and DR5 were not decreased in HBX-Huh-7 cells. These results suggested that the increased expression induced by HBX was not dependent on the activation of JNK pathway. NF-κB pathway was also reported to be responsible for expression of DR4 and DR5 [18, 19]. Furthermore, current researches suggested that activation of NF-κB pathway could be induced by HBX and associated with the abnormality of various biological activities in HBV infection [4, 21–23]. We detected the activation of NF-κB in cells of different groups and found enhanced phosphorylation level of NF-κB in HBX positive cells. Inhibition of NF-κB with BAY11-7082 could reduce the expression of DR5, suggesting that the increased expression of DR5 mediated by HBX was associated with NF-κB-dependent pathway.

With the use of shRNAs against DR4 and DR5, we demonstrated the apoptosis of HBX positive cells mediated by TRAIL was dependant on DR4 and DR5. Moreover, NF-κB pathway that responsible for the upregulation of DR5 was found to contribute to the increase of HBX positive cell apoptosis mediated TRAIL, while inhibition of NF-κB pathway have no significant effect on apoptosis of control cells. Considering the important role of DR4, DR5 and NF-κB pathway in HBX positive cells, targeting these proteins or pathway may be a potential treatment strategy to correct the abnormal biological activity including the cell apoptosis mediated by HBX.

## Conclusion

In summary, we demonstrated that increased apoptosis induced by TRAIL was associated with increased expression of DR5 that mediated by HBX through NF-κB pathway. This finding provided a critical insight into the mechanism of hepatocyte apoptosis mediated by HBX in HBV infection.

## Materials and methods

### Plasmids and reagents

The plasmid pcDNA3.1-X, which contains the full length HBX sequence, and pSilencer3.1-shHBX, a vector for shRNA targeting HBX, were constructed previously [9]. The pGPU6/GFP/Neo plasmids encoding specific shRNAs against DR4 (GCTGTTCTTTGACAAGTTTGC) and DR5 (GCAA GTCTTTACTGTGGAAGA), named as pGPU6/GFP/Neo-DR4 and pGPU6/GFP/Neo-DR5, were purchased from GenePharma Co., Ltd (Suzhou, Jiangsu, China). The pUC18-HBV1.2 plasmid is a gift from Dr. Xiaoben Pan [24]. The rabbit polyclonal antibody against human DR4, and rabbit polyclonal antibody against human DR5 were obtained from Abcam (Cambridge, MA, USA). Recombinant solution human TRAIL was obtained from TeproTech (Rocky Hill, NJ, USA). Mouse anti-HBX polyclonal antibody and Immibilon Western Chemiluminescent HRP Substrate were purchased from Millpore (Billerica, MA, USA). Rabbit polyclonal antibody against JNK, rabbit polyclonal antibody against phosphorylated-JNK (p-JNK), rabbit polyclonal antibody against NF-κB P65, rabbit polyclonal antibody against phosphorylated-NF-κB P65 (p-P65), chemistry inhibitor of JNK (SP600125) and NF-κB (BAY11-7082) were purchased from Sigma-Aldrich (St Louis, MO, USA). Antibodies mouse anti-human β-actin and mouse anti-human Tubulin were purchased from Santa Cruz Biotechnolog (Santa Cruz, CA, USA). G418 was from promega (Madison, WI, USA). Trizol reagent was obtained from Invitrogen (Carlsbad, CA, USA). Annexin V/PI apoptosis kit was from Biovision (Mountain View, CA, USA). UitraTaq enzyme PCR kit was obtained from TIANGEN Biotech (Beijing, China). BCA Protein Assay Kit was from Beyotime Institute of Biotechnology (Jiangsu, China). Goat anti-mouse IgG-HRP, goat anti-rabbit IgG-HRP were purchased from ZSJQ-Bio (Beijing, China). Rabbit anti-HBsAg polyclonal antibody was obtained from Bioss Biotechnology (Beijing, China).

### Cell culture and plasmids transfection

Human hepatocarcinoma cell line Huh-7 cell, obtained from the Cell Bank of Chinese Academy of Sciences, was cultured in DMEM supplemented with 10 % fetal bovine serum (FBS) at 37 °C in 5 % CO2. Huh-7 cells cultured in 6-well plates were transfected with plasmid pcDNA3.1-X (4ug/well), pUC18-HBV1.2 (4ug/well), or cotransfected pUC18-HBV1.2 (4ug/well) with pSilencer3.1-shHBX (2ug/well), using according to the manufacturer’s instructions. Forty-eight hours after transfection, the cells transfected with pcDNA3.1-X were incubated in selection medium containing 0.8 mg/ml G418. After the cells selected with G418 and passed on to 3-5 passages in a month, the expression of HBX mRNA and protein in the resistant clones were detected. The cells steadily expressed with HBX gene and protein were named Huh-7-HBX cell. Huh-7 cells transfected with pcDNA3.1 empty plasmid was as control group. When 48 h post-transfection, these cells transfected with pUC18-HBV1.2 or cotransfected pUC18-HBV1.2 with pSilencer3.1-shHBX plasmids were harvested, and the mRNA and protein levels of target genes were measured by RT-PCR, real-time PCR and western blotting analysis. Huh-7-HBX cells were transfected with DR4 and DR5 shRNA vectors (2ug/well of each) by Lipofectamin2000 as followed the manufacturer’s instructions. At 48 h after transfection, the expression of DR4 and DR5 was tested by real-time PCR and western blotting analysis.

### RT-PCR and real-time PCR analysis

The total RNA of different cells was prepared with Trizol reagent as the manufacturer’s described. The reverse transcription was performed with TIANScript RT Kit. Primer sequences used for detection of HBX were TGTGAAGCTTATGGCTGCTAGGC and TGTGGAATTCTTAGGCAGAGGTG. Primers for HBsAg were CATCTTCTTGTTGGTTCTTCTG and TTAGGGTTT AAATGTATACCC. Primers for β-actin amplificaiton were GGCATCGTGAT GGACTCCG and GCTGGAAGGTGGACAGCGA. The UitraTaq enzyme PCR kit was used for PCR reaction and amplification condition was 94 °C for 5 min, followed with 94 °C for 40 s, 67 °C for 30 s, 72 for 1 min for 30 cycles and a final extension at 72 for 10 min each. The PCR products were subjected to electrophoresis in 1 % agarose gel and visualized by ethidiun bromide staining. The real-time PCR primers for DR4, DR5 AND GAPDH were CTGAGCAACGCAGAC TCGCTGTCCAC and TCAAAGGACACGGCAGAGCCTGTGCCA, GGGAGCCGCTCATGAGGAAGTT GG and GGCAAGTCTCTCTCCCAGCGTCTC, TGGAA GGACTCATGACCACA and TTCAGCTCAGGGATGACCTT, respectively. The real-time PCR was performed with a SYBR master mix (Toyobo, Japan), and amplification conditions were as follows: 94 °C for 3 min, and followed by 40 cycles of 95 °C for 30s, 60 °C for 30 s, 72 for 30s. The relative mRNA expression levels of DR4 and DR5 were normalized based on the GAPDH in each group.

### Western blotting analysis

For protein extracts, cells were lysed in cell lysis buffer with 1 mM phenylmethanesulfonyl fluoride (PMSF). The lysates were collected by scraping from the plates, and then centrifuged at 10,000 g at 4 °C for 10 mins. The protein concentration was measured by BCA Protein Assay Kit and adjusted at equal pace, then total protein was subjected to SDS-PAGE and transferred onto PVDF membranes, the membranes were blocked with 5 % milk in TBS containing 0.01 % Tween-20 for 3 h at room temperature, and incubated with different specific primary antibodies at 4 °C overnight. Then the membranes were incubated with goat anti-mouse IgG-HRP, goat anti-rabbit IgG-HRP separately for 2 h at room temperature. The protein bands were detected with Immibilon Western Chemiluminescent HRP Substrate.

### Flow cytometry analysis

After the cells treated with 100 ng/mL TRAIL for 24 h, 1 × 10^5^ cells were collected, and the cell apoptosis was evaluated by double staining with fluorescein isothiocyanate (FITC)-conjugated Annexin V and propidium iodide (PI) using annexin V/PI apoptosis kit. The flow cytometry analysis for cell apoptosis was described previously (9).

### Statistical analysis

The data were presented as mean ± standard deviation (SD). The comparisons in all experiments were performed using Student’s t-tests or one way ANOVA with SPSS software (version 16.0, SPSS Inc. Chicago, IL, USA). The *p* value < 0.05 was considered statistically significant.

## Abbreviations

DISC: death-inducing signaling complex
DR4: death receptor 4
FITC: fluorescein isothiocyanate
HBV: hepatitis B virus
HBX: HBV X protein
PI: propidium iodide
RT-PCR: reverse transcription polymerase chain reaction
TRAIL: TNF-α related apoptosis inducing ligand
